# Mortality risk of COVID-19 in elderly males with comorbidities: a multi-country study

**DOI:** 10.18632/aging.202456

**Published:** 2020-12-31

**Authors:** Guangdi Li, Yacong Liu, Xixi Jing, Yali Wang, Miao Miao, Li Tao, Zhiguo Zhou, Yuanlin Xie, Yaxiong Huang, Jianhua Lei, Guozhong Gong, Ping Jin, Yuantao Hao, Nuno Rodrigues Faria, Erik De Clercq, Min Zhang

**Affiliations:** 1 Institute of Hepatology and Department of Infectious Diseases, The Second Xiangya Hospital, Central South University, Changsha, China; 2 Hunan Provincial Key Laboratory of Clinical Epidemiology, Xiangya School of Public Health, Central South University, Changsha, China; 3 School of Mathematics and Statistics, Central South University, Changsha, China; 4 The First Hospital of Changsha, Changsha, China; 5 The Fourth Hospital of Changsha, Changsha, China; 6 Department of Endocrinology, The Third Xiangya Hospital, Central South University, Changsha, China; 7 Department of Medical Statistics and Epidemiology, School of Public Health, Sun Yat-sen University, Guangzhou, China; 8 Department of Zoology, University of Oxford, Oxford, UK; 9 Department of Infectious Disease Epidemiology, School of Public Health, Imperial College London, London, UK; 10 Department of Microbiology, Immunology and Transplantation, Rega Institute for Medical Research, KU Leuven, Leuven, Belgium

**Keywords:** COVID-19, SARS-CoV-2, mortality, risk factors, comorbidities

## Abstract

The COVID-19 pandemic causes severe morbidity and mortality. This multi-country study aimed to explore risk factors that drive mortality in COVID-19 patients who received neither dexamethasone nor remdesivir. We analyzed a cohort of 568 survivors and 507 non-survivors from China, European regions, and North America. Elderly males ≥70 years accounted for only 25% of survivors, but this rate was significantly higher in non-survivors from China (55%), European regions (63%), and North America (47%). Compared with survivors, non-survivors had more incidences of comorbidities such as cerebrovascular disease and chronic obstructive pulmonary disease (COPD, p-values<0.05). Survival analyses revealed age, male gender, shortness of breath, cerebrovascular disease, and COPD as mortality-associated factors. Survival time from symptom onset was significantly shorter in elderly versus young patients (median: 29 versus 62 days), males versus females (median: 46 versus 59 days), and patients with versus without comorbidities (mean: 41 versus 61 days). Mortality risk was higher in elderly males with comorbidities than in young females without comorbidities (p-value<0.01). Elderly male survivors with comorbidities also had longer hospital stays than other survivors (25 versus 18.5 days, p-value<0.01). Overall, the high mortality risk in elderly males with COVID-19-associated comorbidities supports early prevention and critical care for elderly populations.

## INTRODUCTION

Coronavirus Disease 2019 (COVID-19) is a global pandemic that is causing significant, widespread increases in morbidity and mortality, while effective antiviral treatments are still under development [1]. As of November 26^th^, 2020, there were more than 60.4 million cases of COVID-19, and the overall case-fatality risk was approximately 2.4% according to an update by the World Health Organization (WHO). With a strong potential of sustained human-to-human transmission, severe acute respiratory syndrome coronavirus 2 (SARS-CoV-2) can quickly spread to vulnerable populations such as elderly individuals with aging-related immune disorders.

Early studies of COVID-19-associated mortality and risk factors have mostly involved small cohorts of non-survivors in Wuhan [2–7]. For instance, COVID-19-associated mortality was observed in 32 (62%) of 52 critically ill patients in Wuhan who received medical care in the intensive care unit [2]. A single-center study reported an increased risk of mortality in COVID-19 patients who were severely or critically ill at hospitalization [3]. Additionally, mortality was observed in both non-severe (1.1%) and severe (32.5%) patients during the 32-day follow-up [4]. A retrospective study of 54 non-survivors showed the increasing odds of death in association with older age, a high sequential organ failure assessment score, and high d-dimer levels at baseline [5]. Another study in Wuhan reported strong associations of older ages with a high risk of acute respiratory distress syndrome and death [6]. Moreover, CD3^+^CD8^+^ T cells and cardiac troponin I were found to be important risk factors based on a small cohort of 21 non-survivors and 158 survivors [7]. Despite the above findings, it remains unclear why some patients are more susceptible to a fatal outcome of COVID-19 and whether elderly patients outside Wuhan harbor unique features with regard to mortality.

To reveal mortality-associated factors, our study collected a large cohort of survivors who recovered from COVID-19 and non-survivors who died of the disease before May 1, 2020. Due to the authorization of remdesivir on May 1 and dexamethasone on June 17 that may affect our results of mortality-related factors, our data collection initially retrieved clinical records of COVID-19 patients who received neither remdesivir nor dexamethasone. By using epidemiological and clinical records of COVID-19 patients from different countries, this multi-country study revealed a high-risk population of elderly patients with specific comorbidities. Given the rapid spread of COVID-19 which is causing high morbidity and mortality worldwide, a special focus on elderly patients with specific comorbidities in current COVID-19 guidelines should be implemented.

## RESULTS

### Epidemiological features

**Figure 1:**
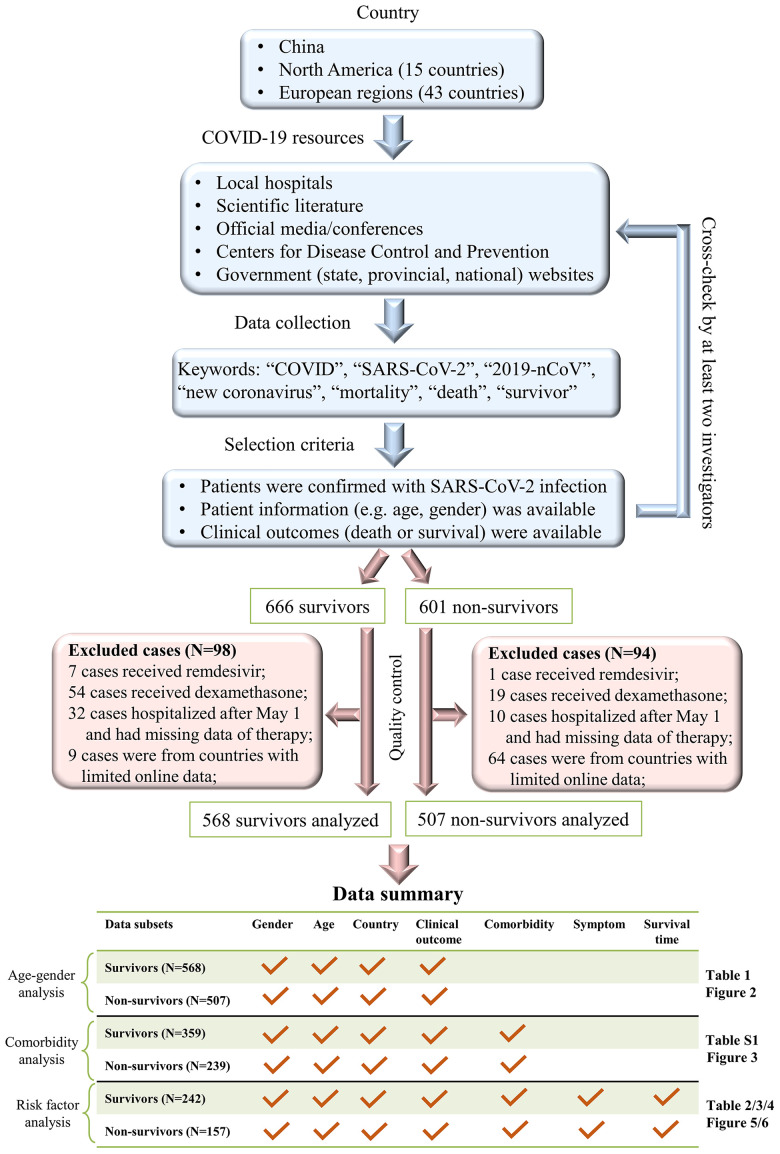
**A workflow of our data collection procedures and dataset summary.**

This study retrieved a multi-country dataset of 1075 COVID-19 patients, including 232 survivors and 183 non-survivors in China, 208 survivors and 258 non-survivors in European regions, and 128 survivors and 66 non-survivors in North America (Table 1). None of the COVID-19 patients received remdesivir or dexamethasone – two approved drugs that may potentially reduce the mortality rate of COVID-19 [8, 9]. All COVID-19 patients were reported between January and April 2020. Our workflow and data summary are provided in Figure 1.

**Table 1:** Basic information of 1075 COVID-19 patients in our study.

		**Sample size**	**Male, n (%)**	**Age, median (IQR)**
Survivors	China	232	108 (47%)	64 (54 to 71)
	European regions	208	117 (56%)	59 (46 to 71)
	North America	128	61 (48%)	46 (39 to 53)
	China	183	117 (64%)	71 (64 to 79)
Non-survivors	European regions	258	169 (66%)	75 (66 to 84)
	North America	66	45 (68%)	71 (56 to 77)

IQR: interquartile range.

**Figure 2:**
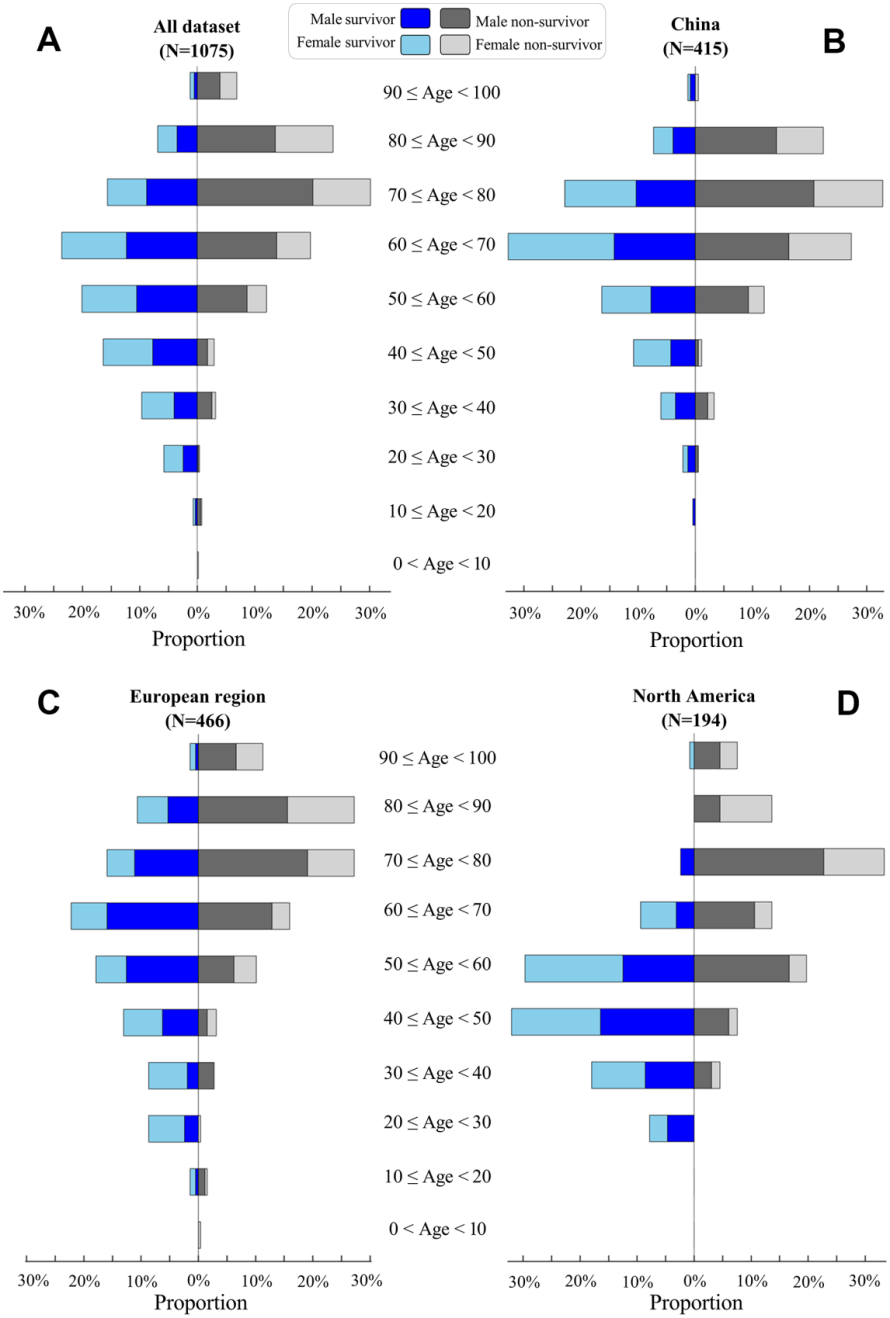
**Age distribution of COVID-19 patients.** Proportions of survivors (left) and non-survivors (right) at different age classes are visualized for all patients (**A**), patients in China (**B**), patients in European regions (**C**), and patients in North America (**D**). For survivors, male and female proportions are visualized by blue and light-blue, respectively. For non-survivors, male and female proportions are visualized by black and gray, respectively.

We analyzed the age distribution of the 1075 patients in our dataset (Figure 2A). The youngest and oldest non-survivors died at 5 and 99 years of age, respectively. COVID-19 survivors were significantly younger than non-survivors in China (median: 64 versus 71 years, p-value<0.001, Figure 2B), European regions (60 versus 75, p-value<0.001, Figure 2C), and North America (46 versus 71, p-value<0.001, Figure 2D). Elderly males ≥70 years accounted for only 25% of 568 survivors, but this rate was significantly higher among non-survivors from China (55%, p-value<0.01), European regions (63%, p-value<0.01), and North America (47%, p-value<0.01).

Our gender analysis suggested that COVID-19 killed more males than females across China, European regions, and North America (Table 1). The proportion of males was significantly higher among non-survivors than survivors in China (64% versus 47%, p-value<0.01), European regions (66% versus 56%, p-value=0.041), and North America (68% versus 48%, p-value=0.01), but such differences were not observed between three sampling origins of non-survivors (p-values>0.05, Table 1).

### Clinical features of survivors and non-survivors

**Figure 3:**
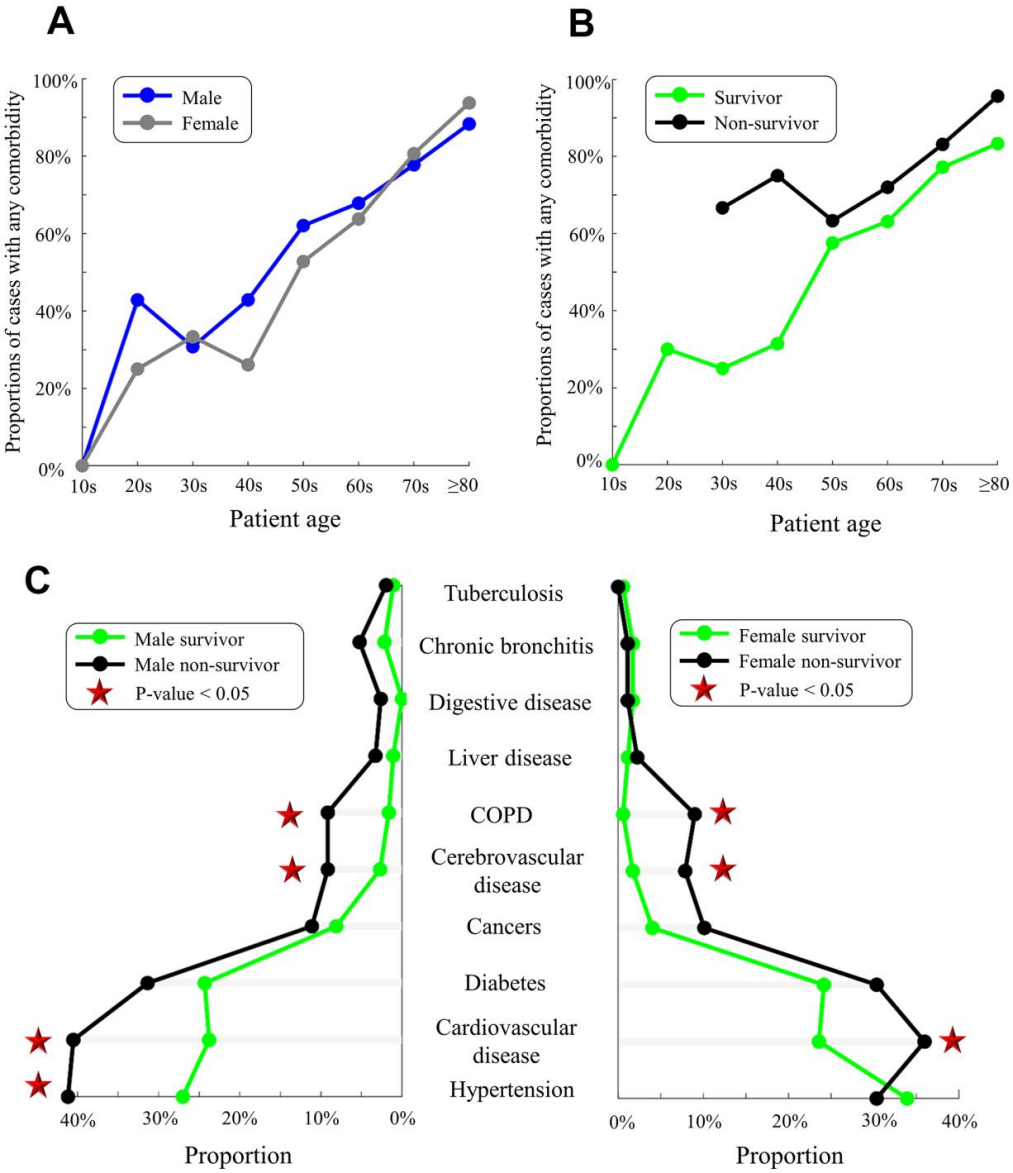
**Pre-existing comorbidities in males/females and survivors/non-survivors.** (**A**) Proportions of males (blue) and females (gray) with any comorbidity. (**B**) Proportions of survivors (green) and non-survivors (black) with any comorbidity. (**C**) Comorbidities in male survivors/non-survivors (left) and female survivors/non-survivors (right). Red stars indicate the significant difference in proportions between survivors and non-survivors (p-value<0.05).

We analyzed the proportions of those with pre-existing comorbidities in a subset of 359 survivors and 239 non-survivors whose comorbidities were recorded (Figure 1). As expected, proportions of comorbidities increased with age in males and females, though comorbidity proportions were slightly higher (but insignificant) in males than in females (Figure 3A and Supplementary Table 1). Moreover, non-survivors had higher proportions of comorbidities than survivors aged from 30 to ≥80 years (Figure 3B). Compared with survivors, non-survivors harbored more comorbidities such as cerebrovascular disease (2% versus 8%, p-value<0.01), cardiovascular disease (24% versus 39%, p-value<0.01), and chronic obstructive pulmonary disease (COPD, 1% versus 9%, p-value<0.01, Supplementary Table 1). In the analysis of comorbidity numbers, more non-survivors had ≥3 comorbidities than survivors (6% versus 16%, p-value=0.01, Supplementary Table 1). Hypertension was highly prevalent among male non-survivors than male survivors (41% versus 27%, p-value=0.006, Figure 3C).

**Figure 4:**
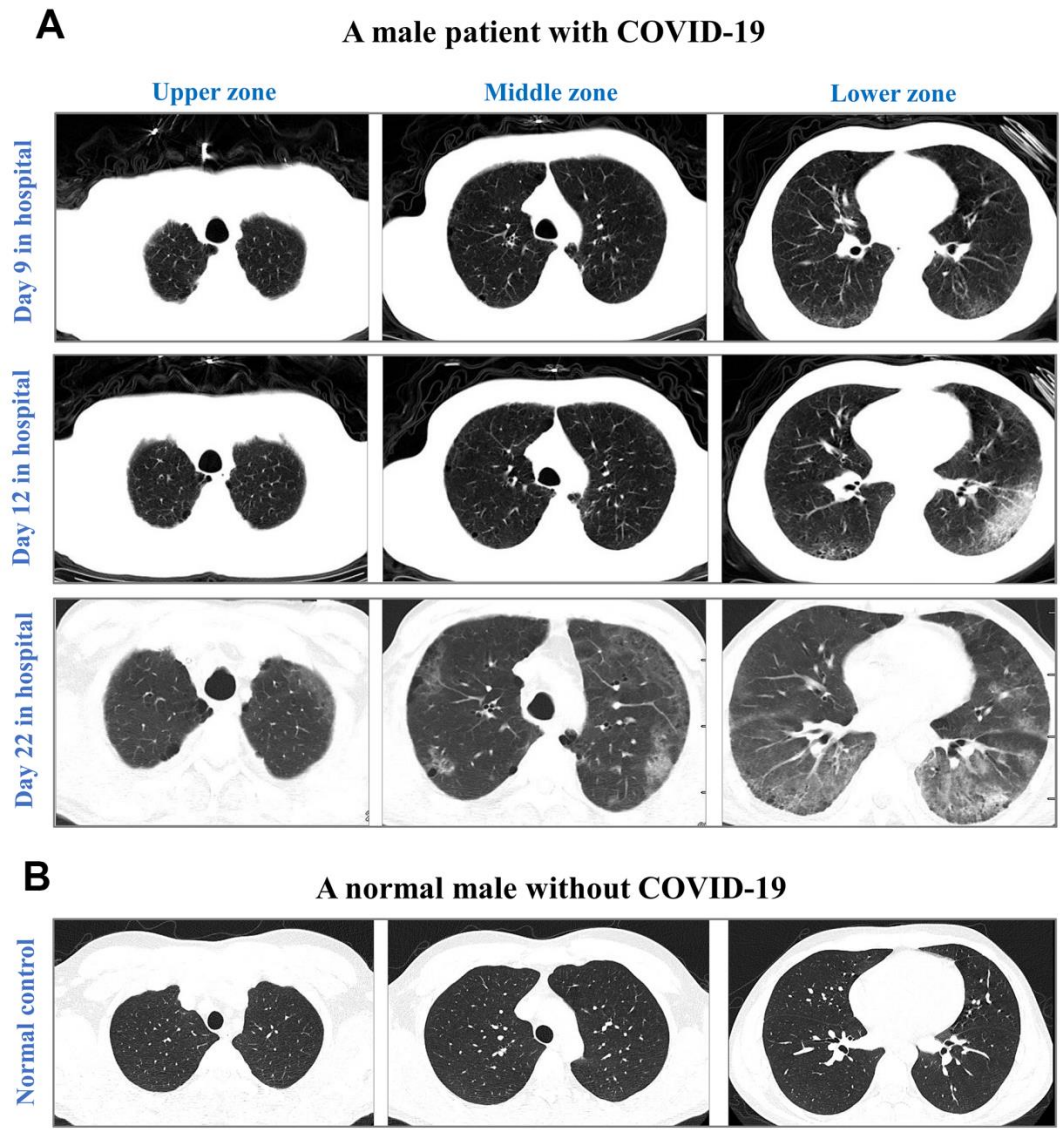
CT images from an elderly male with laboratory-confirmed COVID-19 (**A**) and a normal male without COVID-19 (**B**). CT images were illustrated in the upper zone (above the carina), the middle zone (below the carina up to the inferior pulmonary vein), and the lower zone (below the inferior pulmonary vein). After 9-day hospitalization, CT images showed minor ground-glass opacity (GGO) in subpleural areas of the lower left and right lobes in the COVID-19 male. After 12-day hospitalization, CT images showed progressing GGOs and newly-appeared reticulation. The vascular enlargement was observed in the lesion of the lower left lung. A small amount of bilateral pleural effusion was newly developed. After 22-day hospitalization, CT images showed progressing lesion with multiple newly-appeared GGO in both lungs, predominantly located in subpleural areas of lower lobes. Bronchiectasis of the anterior internal basal segment of the left lower lung was visible. Progressing bilateral pleural effusion was identified. The patient passed away after 24 days of hospitalization in The First Hospital of Changsha.

We next analyzed baseline findings of computed tomography (CT) scans. Incidences of ground-glass opacity, pleural adhesions, and pleural effusion were significantly higher in non-survivors than in survivors (p-values<0.05). Figure 4 highlights the CT results of a non-survivor who had abnormal bilateral lungs with ground-glass opacities and pleural adhesions.

Death-related complications were recorded in a subset of 102 non-survivors. The most common complications were respiratory failure (51%), acute respiratory distress syndrome (28.4%), multiple organ failure (27.5%), shock (22.5%), and hypoxia (13.7%). Males and females had similar incidences of death-related complications (Supplementary Table 2).

### Risk factors associated with COVID-19 mortality

To reveal mortality-associated factors, we examined a subset of 242 survivors and 157 non-survivors whose records contained complete information regarding age, gender, comorbidities, symptoms, clinical outcomes, and survival time from symptom onset to clinical outcomes (Figure 1). As shown in Table 2, the most common symptoms at baseline were fever, cough, shortness of breath, and fatigue (proportions ≥25%). In the comparison of survivors and non-survivors, significant differences in patient age, male gender, shortness of breath, any comorbidity, cerebrovascular disease, cardiovascular disease, chronic liver disease, and COPD were detected (Table 2).

**Table 2:** Clinical features of COVID-19 patients with the complete records of symptoms, comorbidities, clinical outcomes, and survival time.

**Characteristics**	**Total (N=399)**	**Survivors (N=242)**	**Non-survivors (N=157)**	**P-value**
Male	215 (54%)	116 (48%)	99 (63%)	0.003
Age (years)	66 (58 to 74)	65 (55 to 72)	70 (63 to 78)	1.2×10^-5^
Chinese patients	366 (92%)	224 (93%)	142 (90%)	0.45
**Baseline symptoms**				
Any symptom	392 (98%)	236 (98%)	156 (99%)	0.17
Fever	326 (82%)	203 (84%)	123 (78%)	0.16
Cough	256 (64%)	154 (64%)	102 (65%)	0.79
Shortness of breath	149 (37%)	70 (29%)	79 (50%)	1.6×10^-5^
Fatigue	107 (27%)	65 (27%)	42 (27%)	0.98
Chills	63 (16%)	38 (16%)	25 (16%)	0.95
Diarrhea	59 (15%)	42 (17%)	17 (11%)	0.07
Myalgia	58 (14%)	41 (17%)	17 (11%)	0.09
Appetite loss	57 (14%)	39 (16%)	18 (11%)	0.19
Headache	31 (8%)	20 (8%)	11 (7%)	0.65
Nausea	29 (7%)	19 (8%)	10 (6%)	0.58
**Comorbidity**				
Hypertension	163 (41%)	94 (39%)	69 (44%)	0.31
Diabetes mellitus	103 (26%)	56 (23%)	47 (30%)	0.13
Cardiovascular disease	68 (17%)	31 (13%)	37 (24%)	0.005
Cerebrovascular disease	25 (6%)	5 (2%)	20 (13%)	1.7×10^-5^
Cancer	20 (5%)	9 (4%)	11 (7%)	0.14
Chronic bronchitis	16 (4%)	7 (3%)	9 (6%)	0.16
COPD #	11 (3%)	2 (1%)	9 (6%)	0.003
Chronic liver disease	9 (2%)	2 (1%)	7 (5%)	0.017
Digestive disease	8 (2%)	3 (1%)	5 (3%)	0.18
Tuberculosis	6 (2%)	3 (1%)	3 (2%)	0.59
**Any comorbidity**	258 (65%)	141 (58%)	117 (75%)	9.0×10^-4^
1 comorbidity	136 (34%)	80 (33%)	56 (36%)	
2 comorbidities	78 (20%)	47 (19%)	31 (20%)	0.004
≥3 comorbidities	44 (11%)	14 (5%)	30 (12%)	
**Time to clinical outcome**				
Symptom onset to outcome	29 (19 to 42)	38 (27 to 47)	17 (12 to 27)	<0.01
Length of hospital stay	17 (10 to 27)	20 (11 to 28)	11 (7 to 21)	2.2×10^-10^

#: COPD: chronic obstructive pulmonary disease.

We next used Cox proportional hazards models to evaluate time-dependent hazards of baseline factors for the clinical outcome of death. Univariate Cox regression analyses revealed seven risk factors: age, male gender, shortness of breath, any comorbidity, cerebrovascular disease, cardiovascular disease, and COPD (p-values<0.05, Table 3). Multivariate Cox regression analyses further confirmed five significant factors: male gender (hazard ratio (HR): 1.41, p-value=0.039), age (HR: 1.03, p-value<0.01), shortness of breath (HR: 1.74, p-value=0.0008), cerebrovascular disease (HR: 3.28, p-value<0.01), and COPD (HR: 2.19, p-value=0.03, Table 3). These five factors with the effect size of Cohen’s d ≥0.4 remained significant in the propensity score-matched samples that potentially reduced the confounding effect (Table 4).

**Table 3:** Risk factors in the survival model of COVID-19 using Cox proportional hazards models.

	**Univariate analysis**		**Multivariate analysis**	
	**HR (95% CI)**	**P-value**	**HR (95% CI)**	**P-value**
Male	1.53 (1.29 to 1.80)	0.01	1.41 (1.19 to 1.67)	0.039
Patient age*	1.04 (1.03 to 1.05)	6.4×10^-8^	1.03 (1.02 to 1.04)	3.0×10^-5^
Region ^&^	0.52 (0.39 to 0.68)	0.06		
**Signs or symptoms**				
Any symptom	2.65 (0.97 to 7.23)	0.33		
Fever	0.76 (0.63 to 0.93)	0.16		
Cough	0.89 (0.75 to 1.05)	0.49		
Shortness of breath	1.86 (1.59 to 2.18)	1.0×10^-4^	1.74 (1.47 to 2.05)	0.0008
Fatigue	0.90 (0.75 to 1.08)	0.55		
Myalgia	0.64 (0.49 to 0.82)	0.08		
Chills	0.92 (0.74 to 1.14)	0.69		
Diarrhea	0.63 (0.48 to 0.81)	0.07		
Headache	0.81 (0.59 to 1.11)	0.49		
Nausea	0.78 (0.56 to 1.09)	0.46		
Appetite loss	0.64 (0.50 to 0.82)	0.08		
**Comorbidity**				
Any comorbidity	1.79 (1.49 to 2.15)	0.002		
Hypertension	1.23 (1.05 to 1.45)	0.19		
Diabetes mellitus	1.27 (1.06 to 1.51)	0.18		
Cardiovascular disease	1.74 (1.44 to 2.10)	0.004		
Cerebrovascular disease	4.11 (3.23 to 5.24)	5.1×10^-9^	3.28 (2.55 to 4.23)	2.9×10^-6^
Chronic liver disease	1.90 (1.29 to 2.80)	0.09		
Digestive disease	1.93 (1.23 to 3.05)	0.15		
Cancer	1.50 (1.10 to 2.05)	0.20		
Chronic bronchitis	1.76 (1.25 to 2.48)	0.10		
COPD	3.45 (2.44 to 4.88)	3.6×10^-4^	2.19 (1.53 to 3.15)	0.03
Tuberculosis	1.28 (0.71 to 2.29)	0.68		

* : Increase per year. &: Patients in China versus Europe/North America.

**Table 4:** Mortality-associated factors in the original sample and the propensity score-matched samples.

	**Original sample**				
	**HR (95% CI)**	**P-value**	**OR (95% CI)**	**P-value**	**Cohen’s d**
Male	1.53 (1.29 to 1.80)	**0.01**	1.85 (1.23 to 2.80)	0.003	0.339
Patient age	1.04 (1.03 to 1.05)	**6.4×10^-8^**	-	-	0.586
Shortness of breath	1.86 (1.59 to 2.18)	**1.0×10^-4^**	2.49 (1.64 to 3.78)	1.9×10^-5^	0.503
Any comorbidity	1.79 (1.49 to 2.15)	0.002	2.20 (1.42 to 3.42)	4.3×10^-4^	0.435
Cardiovascular disease	1.74 (1.44 to 2.10)	0.004	2.10 (1.24 to 3.56)	0.006	0.409
Cerebrovascular disease	4.11 (3.23 to 5.24)	**5.1×10^-9^**	6.92 (2.54 to 18.85)	1.6×10^-4^	1.066
COPD	3.45 (2.44 to 4.88)	**3.6×10^-4^**	7.30 (1.56 to 34.24)	0.01	1.096
	Matched sample*				
	**HR (95% CI)**	**P-value**	**OR (95% CI)**	**P-value**	**Cohen’s d**
Male	1.76 (1.19 to 2.62)	**0.005**	2.07 (1.28 to 3.34)	0.003	0.401
Patient age	1.07 (1.04 to 1.09)	**1.2×10^-7^**	-	-	0.859
Shortness of breath	6.92 (3.8 to 12.77)	**6.1×10^-10^**	11.20 (5.65 to 22.20)	4.4×10^-12^	1.332
Any comorbidity	1.00 (0.63 to 1.56)	0.99	1.08 (0.63 to 1.85)	0.78	0.042
Cardiovascular disease	1.48 (0.82 to 2.67)	0.19	1.79 (0.84 to 3.80)	0.13	0.321
Cerebrovascular disease	52.3 (6.7 to 407.9)	**1.6×10^-4^**	96.0 (10.4 to 890.6)	5.9×10^-5^	2.516
COPD	4.93 (1.01 to 24.1)	**0.049**	12.3 (1.3 to 113.1)	0.03	1.381

*: The propensity score-matched samples are described in Supplementary Table 3.1 to Supplementary Table 3.7.

-: The OR of patient age was not measured because patient age was a continuous variable.

**Figure 5:**
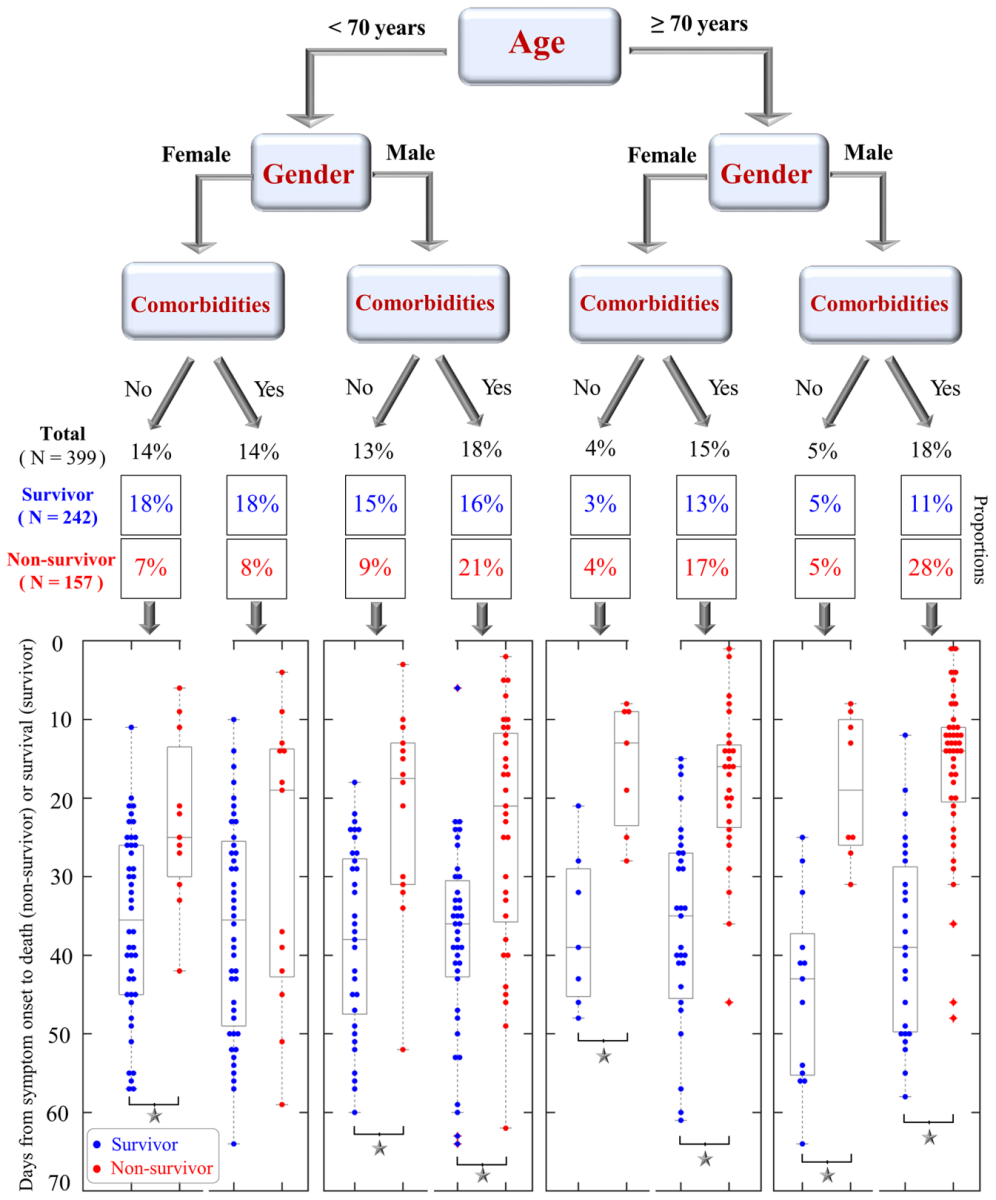
**A tree model that shows the proportions of COVID-19 survivors and non-survivors based on three conditions: patient age (<70 or ≥70 years), gender (female, male), and pre-existing comorbidities (yes or no).** Scatter plots at the bottom visualize the days from symptom onset to clinical outcomes (death for non-survivors and survival for survivors). Blue and red dots are shown for survivors and non-survivors, respectively. Significant differences in survival time between survivors and non-survivors are indicated by black asterisks.

A tree model was built in Figure 5 to visualize proportions of survivors and non-survivors based on 8 possible conditions of three pre-existing factors at baseline: age (<70 or ≥70 years), gender (male or female), and any comorbidity (yes or no). Among the 8 possible conditions, the highest proportion of mortality was observed in elderly males with comorbidities (28%, 44/157), whereas the highest proportion of survivors was found in young females without comorbidities (18%, 44/242). A higher proportion of non-survivors was often found among males, those ≥70 years, and/or those with comorbidities than among females, those <70 years, and/or those without comorbidity (Figure 5). Similar patterns were also observed in the tree models of age, gender plus either cerebrovascular disease or COPD (Supplementary Figure 1).

### Impact of risk factors on survival time and length of hospital stay

**Figure 6:**
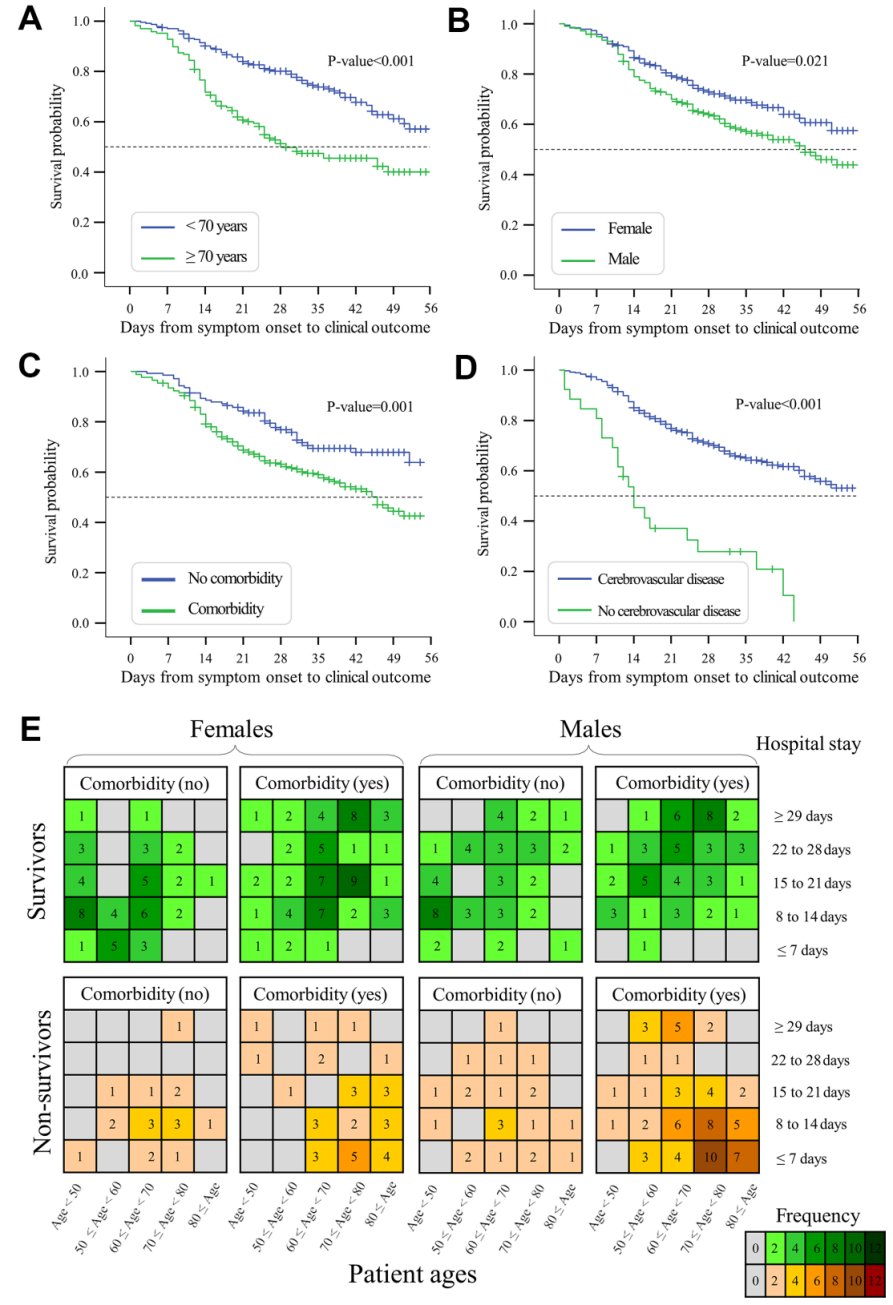
**Kaplan-Meier curves and plots of hospital stays.** (**A**) Survival probability of elderly patients ≥70 years versus young patients <70 years. (**B**) Survival probability of males versus females with COVID-19. (**C**) Survival probability of patients with versus without any comorbidity. (**D**) Survival probability of patients with versus without cerebrovascular disease. (**E**) Length of hospital stays under the conditions of patient age, gender, and comorbidities. Colored cells indicate the number of observed cases regarding the length of hospital stay. Days from symptom onset to clinical outcomes are shown in Supplementary Figure 3.

Kaplan-Meier curves were evaluated to understand whether risk factors such as age, gender, and comorbidities affect survival time from symptom onset to death - the primary endpoint of clinical outcomes. As shown in Figure 6A, the median survival time of elderly patients ≥70 years was significantly shorter than that of patients <70 years (median: 29 versus 62 days, p-value<0.001). Male patients had a shorter survival time than female patients (median: 46 versus 59 days, p-value=0.021, Figure 6B). The survival time of patients with comorbidities was shorter than those without any comorbidity (mean: 41 versus 61 days, p-value=0.001, Figure 6C). Furthermore, the survival time of patients with ≥3 comorbidities (mean: 27, IQR: 20 to 33 days) was shorter than those with 1 or 2 comorbidities (mean: 43, IQR: 40 to 46.5 days) and those without any comorbidity (mean: 60, IQR: 55 to 66 days, p-values<0.01, Supplementary Figure 2A). Patients with cerebrovascular disease (median: 14 versus 59 days, p-value<0.001, Figure 6D) or COPD (median: 11 versus 59 days, p-value<0.001, Supplementary Figure 2B) also had a shorter survival time than patients without these comorbidities.

We next evaluated the length of hospital stay under pre-existing conditions of age, gender, and comorbidities (Figure 6E). The length of hospital stay was shorter among non-survivors (median: 11, interquartile range (IQR): 7 to 21 days) than among survivors (median: 20, IQR: 11 to 28 days, p-value<0.01). The median time from hospital admission to discharge was significantly longer in elderly survivors (median: 23, interquartile range (IQR): 18.5 to 32 days) than in young survivors (median: 17, IQR: 10 to 23 days, p-value<0.001). Survivors with comorbidities had a longer period of hospital stay than survivors without comorbidities (median: 21 versus 15 days, p-value<0.001). For survivors, elderly males with comorbidities required 25 days (IQR: 18 to 35 days) of hospital stay, which was much longer than young females without comorbidities (median: 14, IQR: 9 to 19 days, p-value<0.001). For non-survivors, the median time from hospital admission to death was 11.5 days (IQR: 7 to 22 days). Moreover, elderly non-survivors had a shorter length of hospital stay compared with young non-survivors (median: 10 versus 14 days, p-value=0.003). The length of hospital stay was shorter in elderly male non-survivors (median: 10, IQR: 6 to 18.5 days) than in young male non-survivors (median: 15, IQR: 9.5 to 27 days, p-value=0.003). Overall, mortality-associated risk factors exert an impact on the hospital stay and survival time in COVID-19 patients.

## DISCUSSION

To understand why some COVID-19 patients are more susceptible to fatal outcomes, we explored epidemiological and clinical records of survivors and non-survivors from China, European regions, and North America. Our study revealed three major findings. (i) High mortality risk of COVID-19 was consistently found among elderly males from China, Europe, and North America. (ii) Risk factor and survival analyses revealed mortality-associated risk factors of age, male gender, shortness of breath, and chronic comorbidities such as cerebrovascular disease and COPD. (iii) Mortality-associated risk factors exert an impact on the survival time and hospital stays of elderly patients. To reduce mortality rates and hospital burden, we carried out a multi-country study on COVID-19-induced mortality, and the results support the early prevention and critical care of elderly males with specific comorbidities.

### Elderly adults with an increased risk of COVID-19

In agreement with previous findings [2, 4, 10, 11], our survival analyses suggested that the mortality risk of COVID-19 increased with patient age (HR: 1.04 increase per year), and most deaths were observed in elderly adults, especially those ≥70 years (>50% of non-survivors). Of interest, the high mortality risk of elderly adults was consistently observed in China, European regions, and North America (Figure 2). Indeed, similar findings have been consistently reported by single-country studies of COVID-19 patients in many countries such as America [12], Belgium [13], Canada [14], France [15], Germany [16], Italy [17], Mexico [18], Poland [19], Romania [20], Russia [21], Spain [22], and the United Kingdom [23]. Of note, age, gender, and chronic comorbidities are key factors that determine hospitalization rather than outpatient care [10]. For instance, elderly survivors required a longer length of hospital stay than non-survivors (Figure 6). The elderly required longer hospital stays than young adults, probably because close monitoring of their recovery requires more time in the hospital. Similar to our findings, a small-cohort study of 17 non-survivors reported a short time (median: 11.5 days) from symptom onset to death in patients ≥70 years [24]. Overall, our study and literature findings support the early prevention of COVID-19 in elderly adults due to their high risk.

Why are elderly adults more vulnerable to COVID-19? Although the exact mechanisms remain unclear, several hypotheses have been proposed [25]. First, compared with young patients, elderly adults with compromised immunity might have slower, less coordinated, and less efficient immune responses to COVID-19 [26, 27]. Additionally, the innate and adaptive systems are possibly suboptimal in elderly patients. For instance, T and B lymphocytes are critical components of adaptive immune responses to emerging infections, but elderly patients may experience age-related dysfunction with a decreased production of T and B lymphocytes [28]. Second, elderly adults may have an increased risk of exposure to COVID-19, problems with access to health services, and less opportunity to receive respiratory support in resource-limited settings [29]. Third, elderly adults usually have comorbid conditions such as acute respiratory distress syndrome, which may diminish their recovery from COVID-19 [30].

### Elderly males with a high mortality risk

In agreement with previous studies [12–23], our study showed gender disparities in that COVID-19 killed more males than females especially in elderly populations (Figure 2), and our survival analyses revealed age and male gender as risk factors to be associated with mortality (Table 3). Of note, the fatality rate was higher in males (2.84%, 653/22981) than females (1.71%, 370/21691) in mainland China [31]. In Italy, males accounted for 82% of 1591 critically ill patients with ICU admission [32]. During the screening of COVID-19 in Iceland, a higher proportion of positive cases was observed in males (1.1%, 46/5004) than females (0.7%, 41/5793) [33]. Accumulated evidence shows that more males than females are dying of COVID-19 across more than 100 countries according to sex-disaggregated data from Global Health 50/50 (http://globalhealth5050.org/covid19/).

Why does COVID-19 kill more males than females? Although the exact mechanisms remain unknown, several hypotheses have been proposed to explain gender disparities. (i) The X chromosome encodes the human receptor angiotensin-converting enzyme 2 (ACE2, gene ID: 59272), which is the human receptor mediating SARS-CoV-2 entry into human cells. Expression of ACE2 might vary in different populations due to genetic differences [34]. (ii) Males and females have different immune responses to COVID-19 due to the sex chromosomes (XY in males, XX in females), sex hormones (androgens, estrogens), and regulatory genes associated with the immune system [35]. Since the X chromosome encodes the largest number of immune-related genes in the human genome, females carrying two copies of the X chromosome may have immunological advantages regarding resistance to viral pathogens, but they also develop more autoimmune diseases [36, 37]. (iii) Different socioeconomic and lifestyle behaviors such as smoking, alcohol consumption, and personal hygiene may also play a role in gender differences in susceptibility to COVID-19 [38].

### Comorbidities increase the risk of COVID-19

In agreement with previous studies [39–41], we observed a higher proportion of comorbidities (e.g. cerebrovascular disease, cardiovascular disease, COPD) in non-survivors than in survivors (Table 2). Furthermore, pre-existing comorbidities may reduce the survival time in non-survivors and increase the length of hospital stay of survivors (Figure 6). Similar to our results in Table 2, severely ill patients had more comorbidities, including hypertension (56%), heart disease (21%), diabetes (18%), cerebrovascular disease (12%), and cancer (7%) [42]. Comorbidities were also observed in 94% of 5700 COVID-19 patients hospitalized in New York [43]. Our study showed slightly higher (but insignificant) proportions of comorbidities such as diabetes mellitus, cardiovascular disease, cerebrovascular disease, chronic bronchitis, and COPD in males than females (Supplementary Table 1). Multisystem organ dysfunction in males might be associated with a high rate of mortality [32].

What are the impacts of comorbidities on the morbidity and mortality of COVID-19? Although the possible mechanisms are still under investigation, answers likely depend on the type of comorbidity. First, our analyses revealed that the comorbidities of cerebrovascular disease and COPD are time-dependent factors associated with a fatal outcome of COVID-19 (Tables 3, 4). Of interest, cerebrovascular disease was also recognized as a risk factor in a retrospective study of 50 non-survivors and 1540 survivors [40]. Furthermore, a meta-analysis reported an approximately 2.5-fold increase in the odds of severe disease in the presence of cerebrovascular disease [44]. Pre-existing COPD was previously confirmed as a key factor that contributes to the worse progression and outcome of COVID-19 [45]. Due to SARS-CoV-2 invasion of the central nervous system, severely ill patients are more likely to develop neurologic manifestations, especially acute cerebrovascular disease [46]. Second, possible crosstalk between diabetes and COVID-19 might be involved with ACE2 and dipeptidyl peptidase-4, two important human proteins in the biological pathways of both diseases [47, 48]. Third, cardiovascular disease involves an increased risk of in-hospital death in COVID-19, which might be mediated by ACE2-dependent myocardial infection [49] or the high inflammatory burden that can induce vascular inflammation, myocarditis, and cardiac arrhythmias [50]. Fourth, patients with lung cancer, hematological cancer, or cancers in metastatic stages have higher rates of severe outcomes than patients without cancer [51]. Overall, comorbidities are likely associated with the severity of COVID-19 [39, 41].

### Limitations

First, we initially intended to collect all available online records of COVID-19 cases worldwide, but detailed medical records of individual patients were mostly lacking probably because the privacy of patient data is protected and detailed records are often not published in an emergent outbreak. Nevertheless, our major findings of elderly males at high risk for COVID-19 have been consistently confirmed by single-country studies [12–23]. Future multi-country studies are yet needed to retrieve complete records worldwide and characterize COVID-19 mortality from a global perspective. Second, our retrospective study describes potential associations of risk factors with mortality, but crosstalk between human and viral proteins in the lifecycle of SARS-CoV-2 needs to be clarified by future studies. Third, the role of treatments in fatal outcomes of COVID-19 was not analyzed because multiple treatments (e.g. antiviral/antibiotic/antifungal therapies, ventilation, oxygen therapy) are often administered. The possible role of prevention strategies in reducing mortality was not analyzed due to limited data. Moreover, controlled cohorts were not available for our study. Randomized controlled cohorts are thus needed to assess the use of potential treatments in elderly patients with COVID-19.

## CONCLUSIONS

Given the rapid spread of COVID-19 worldwide, a better understanding of high-risk populations is important for decision making in the treatment and prevention of the disease. The clinical features of non-survivors inside and outside Wuhan are similar [28], supporting universal strategies to handle COVID-19 across different cities and countries. As of today, there is no cure to eliminate COVID-19 so that prevention strategies are critical for reducing COVID-19 infections. In addition to prevention measures (e.g. face masks, good hygiene, social distancing, contract tracing, travel restrictions, lockdowns, and quarantine) that limit the spread of COVID-19, intensive surveillance and hospital/outpatient caregiving should be adapted for elderly patients with COVID-19. Current guidelines should also pay more attention to high-risk populations of elderly patients with COVID-19-associated comorbidities, complications, and polypharmacy.

## MATERIALS AND METHODS

### Data collection of COVID-19 patients

Our workflow, as shown in Figure 1, was conducted to collect clinical records for COVID-19 patients in China, North America, and European regions. First, we collected epidemiological and clinical records of COVID-19 patients who were hospitalized before May 1^st^, 2020, in the Sino-French New-City Tongji Hospital in Wuhan. At hospital admission, all patients were diagnosed with COVID-19. Nasal and pharyngeal swab specimens were collected to assess the presence of SARS-CoV-2 using real-time RT-PCR tests according to WHO interim guidelines. All survivors, who fulfilled discharge criteria, were discharged for 14-day home quarantine based on the New Coronavirus Diagnosis and Treatment Guidelines in China (Supplementary Method 1). A total of 232 survivors and 60 non-survivors were included in our dataset. Second, to increase the sample size, we extracted clinical records for all COVID-19 non-survivors from The First Hospital of Changsha in China. Third, we attempted to extract online records for COVID-19 individuals in China, European regions, and North America. Online collections were conducted using a four-fold procedure. (i) We searched online records of COVID-19 patients in China, 15 countries in North America, and 43 countries in Europe (Supplementary Table 4) where a large number of COVID-19 patients were reported between January and April 2020. (ii) We searched records of COVID-19 individuals in the scientific literature, official media/conferences, hospital websites, government (state, provincial, national) websites, and Centers for Disease Control and Prevention. Only individual cases with epidemiological and clinical information were retrieved, while clinical studies or meta-analyses that shared no individual records were not considered. (iii) We used keywords such as: “COVID”, “SARS-CoV-2”, “2019-nCoV”, “new coronavirus”, “mortality”, “death” and “survivor” to screen online data. (v) Duplicates were removed and all records were cross-checked by at least two investigators.

The data extraction (as described above) retrieved a total of 1267 patients, including 666 survivors and 601 non-survivors. Because remdesivir and dexamethasone significantly reduce COVID-19 mortality [8, 9], our analyses excluded patients who received remdesivir (N=8) and/or dexamethasone (N=73). Moreover, 42 patients were excluded because they had no treatment records and were hospitalized after May 1, 2020. Of 59 countries in our search list, 35 had fewer than five extracted records in our dataset, which is unlikely to be representative; therefore, 73 cases from these countries were excluded. Supplementary Table 4 summarizes the country list and case numbers in 59 countries. For data transparency, the compiled online resources of 981 COVID-19 patients are shared in Supplementary Data 1.

A total of 568 survivors and 507 non-survivors with basic information regarding age, gender, country, and clinical outcomes were included in our age-gender analysis (Table 1 and Figure 2). Additional information on baseline symptoms and comorbidities was also recorded in a subset of 359 survivors and 239 non-survivors, which was used for comorbidity analyses (Figure 3). Survival time, as defined by the days from symptom onset of clinical outcomes, was identified in a subset of 242 survivors and 157 non-survivors and, subsequently used for risk-factor analyses and survival analyses (Tables 2–4 and Figures 5, 6). Figure 1 summarizes our workflow and data structures.

### Data management

Our datasets included demographic features (age, gender, cities), signs and symptoms, pre-existing comorbidities, complications before death, and other available data (e.g. symptom onset date, admission date, computerized tomography data) for COVID-19 survivors and non-survivors. Symptoms, comorbidities, and complications were treated as categorical variables (yes or no). Clinical outcomes were defined by the status of death (non-survivors) or hospital discharge after the clearance of COVID-19 (survivors). For non-survivors, the survival time was defined by days from symptom onset to the date of death. Since death was not observed before hospital discharge, survivors were treated as censored data in which the observation time was defined by days from symptom onset to hospital discharge.

### Statistical analysis

In comparisons of COVID-19 survivors and non-survivors, continuous variables were analyzed using Mann-Whitney U tests, while categorical variables were analyzed using χ^2^ tests or Fisher’s exact tests as appropriate. Survival analyses were performed using Cox proportional hazards models. Hazard ratios with the 95% confidence interval were assessed to reveal the effects of risk factors on survival-time outcomes: either death for non-survivors or hospital discharge for survivors. Kaplan-Meier curves were built to show survival time among patient groups (e.g. elderly versus young patients), and significant differences were examined by log-rank tests. A common approach called pairwise deletion was applied to handle missing data. Since no random sampling was conducted, all statistical analyses were descriptive. To reduce the confounding effect in this nonrandomized study, the propensity-score matching method in SPSS V26.0 was applied with default settings (e.g. one-to-one matching, matching tolerance: 0.02) to assess whether any observed confounding was adequately reduced. The effect size of risk factors was estimated by Cohen's d [52]. In all significance tests, two-tailed tests were performed and P-values <0.05 were considered statistically significant.

### Ethics declarations

This retrospective study was performed in accordance with the Helsinki Declaration and was approved by the Ethics Committees of The Second Xiangya Hospital, Central South University (ID: LYF2020060), and The First Hospital of Changsha (ID: KX2020002). Written informed consent was waived for analyzing archived records in this retrospective study.

### Availability of clinical data

Supplementary Data 1 shares online records of COVID-19 patients.

## Supplementary Material

Supplementary Figures

Supplementary Tables

Supplementary Method 1

Supplementary Dataset 1
