# Supplementary material for: Mortality risk of COVID-19 in elderly males with comorbidities: a multi-country study
Source: Aging (Albany NY). 2020 Dec 31;13(1):27–60. doi: 10.18632/aging.202456 (PMC7835001; doi:10.18632/aging.202456)
Supplement: Supplementary Figures [file aging-13-202456-s001.pdf]

SUPPLEMENTARY FIGURES

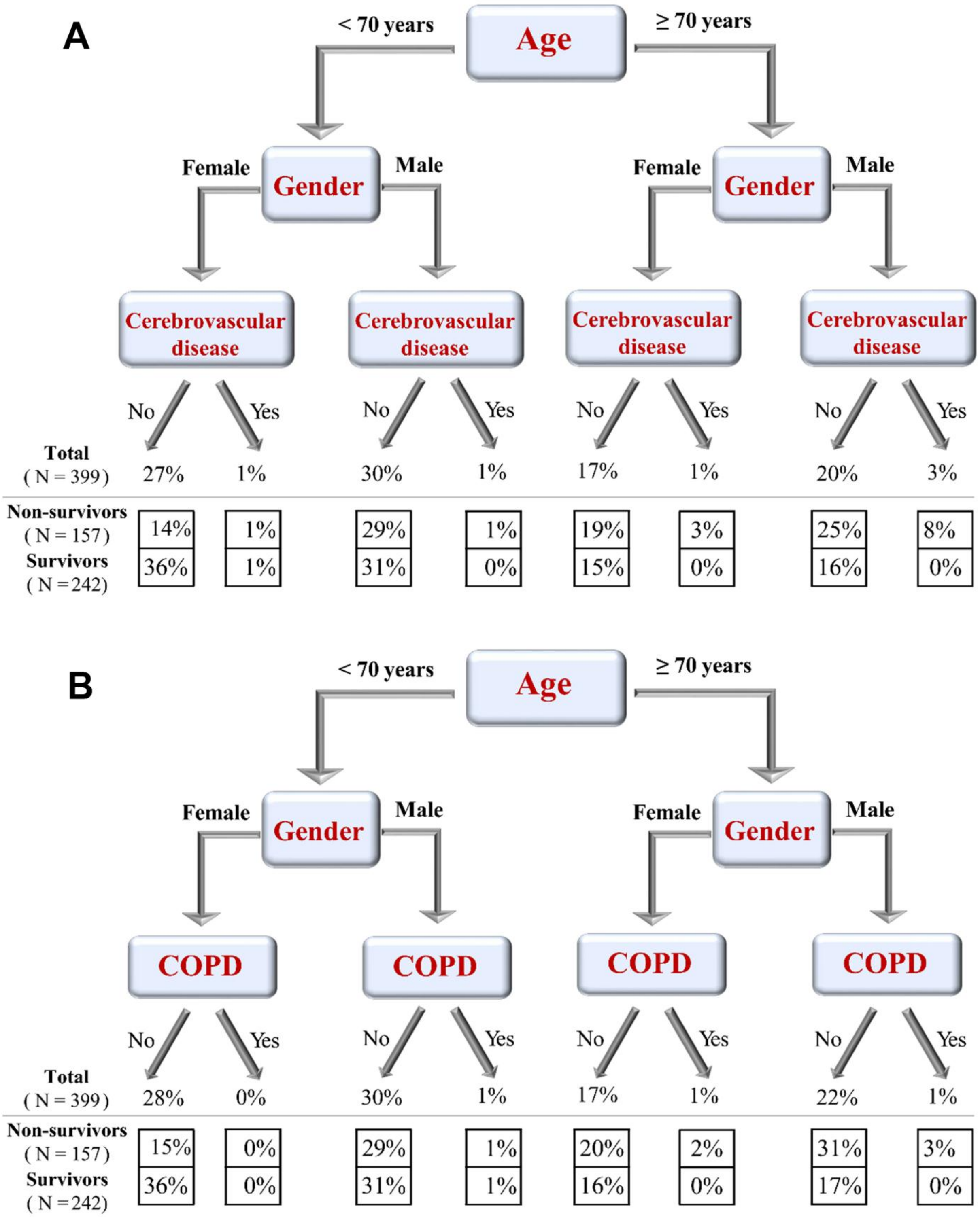

Supplementary Figure 1. A tree model shows proportions of COVID-19 survivors and non-survivors based on patient age and gender plus either cerebrovascular disease (**A**) or COPD (**B**).

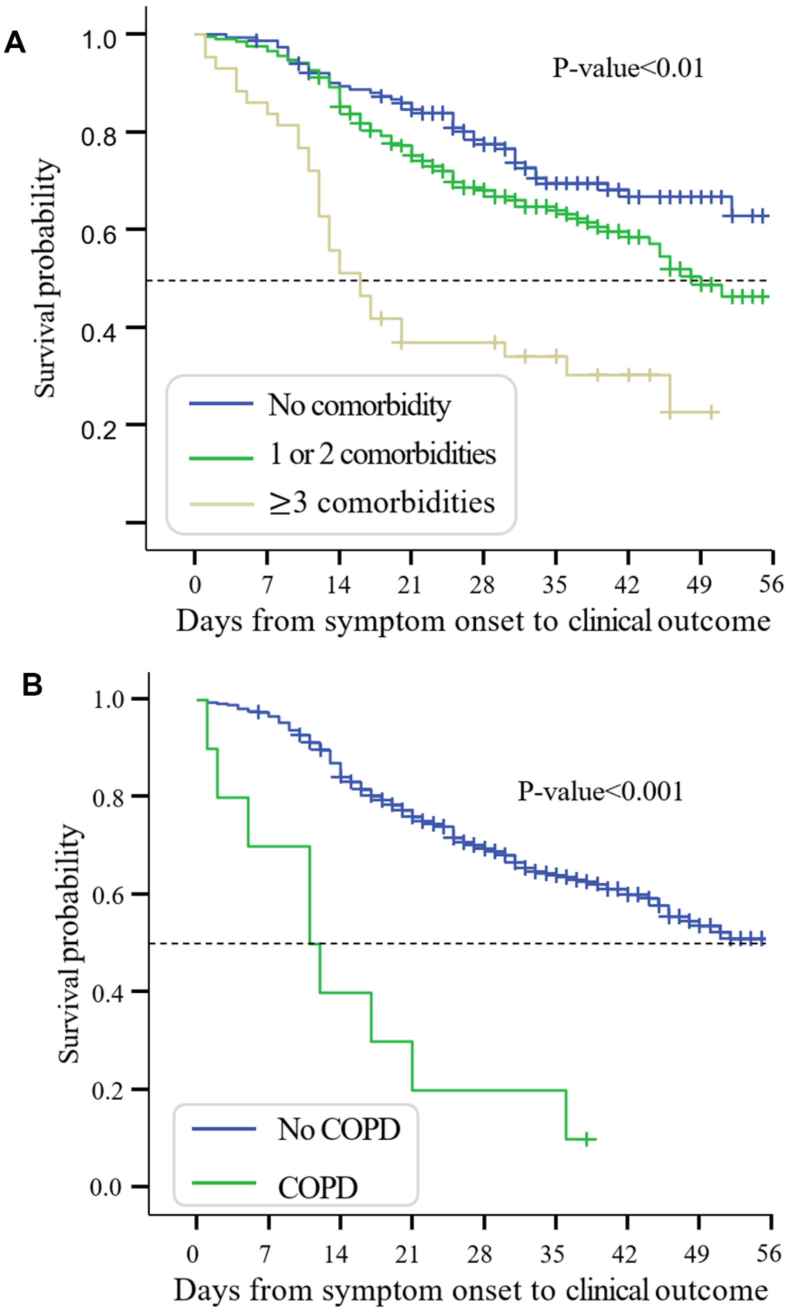

Supplementary Figure 2. Kaplan-Meier curves of COVID-19 patients. (**A**) Kaplan-Meier curves of COVID-19 patients with ≥3 comorbidities (brown), 1 or 2 comorbidities (green), or without any comorbidity (blue). (**B**) Kaplan-Meier curves of COVID-19 patients with or without COPD. Survival probabilities of patients with COPD (green) and without COPD (blue) are shown with the significant difference (p-value < 0.001).

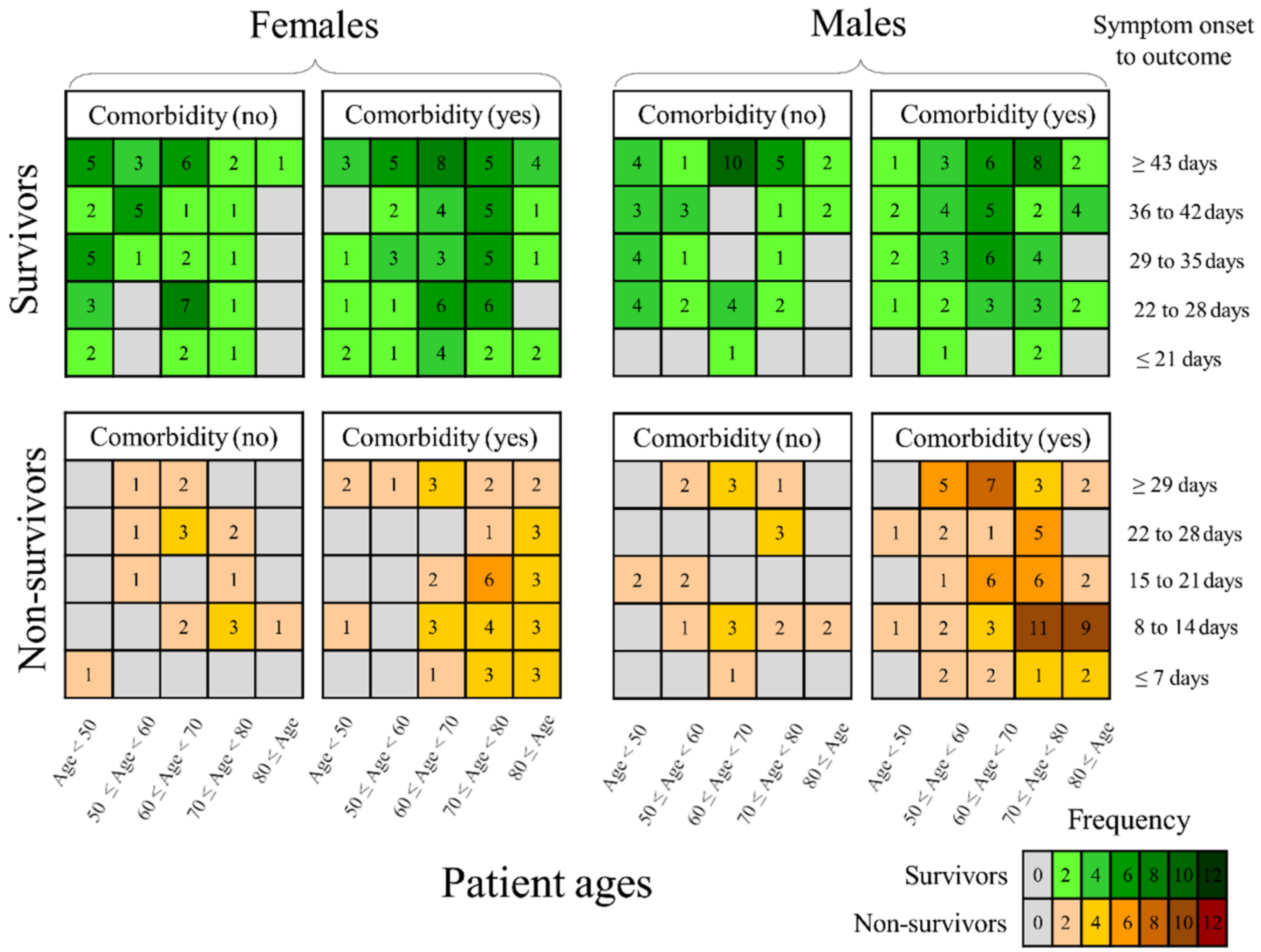

Supplementary Figure 3. Days from symptom onset to clinical outcomes under the conditions of patient age, gender, and comorbidities.
