# Supplementary material for: Mortality risk of COVID-19 in elderly males with comorbidities: a multi-country study
Source: Aging (Albany NY). 2020 Dec 31;13(1):27–60. doi: 10.18632/aging.202456 (PMC7835001; doi:10.18632/aging.202456)
Supplement: Supplementary Tables [file aging-13-202456-s002.pdf]

SUPPLEMENTARY TABLES

Supplementary Table 1. Proportions of comorbidities in females/males and survivors/non-survivors.

	Total (N=598)	Survivors (N=359)	Non-survivors (N=239)	P-value	Females (N=338)	Males (N=260)	P-value
Any comorbidity	412 (69%)	218 (61%)	194 (81%)	**<0.01**	173 (67%)	239 (71%)	0.27
1 comorbidity	209 (35%)	122 (34%)	87 (36%)		95 (37%)	114 (34%)	
2 comorbidities	142 (24%)	73 (20%)	69 (29%)	**0.016**	58 (22%)	84 (25%)	0.20
≥3 comorbidities	61 (10%)	23 (6%)	38 (16%)		20 (8%)	41 (12%)	
Hypertension	198 (33%)	109 (30%)	89 (37%)	0.08	85 (33%)	113 (33%)	0.85
Diabetes mellitus	161 (27%)	87 (24%)	74 (31%)	0.07	68 (26%)	93 (28%)	0.71
Cardiovascular disease	178 (30%)	85 (24%)	93 (39%)	**<0.01**	72 (28%)	106 (31%)	0.33
Cancers	47 (8%)	22 (6%)	25 (11%)	0.05	15 (6%)	32 (10%)	0.10
Cerebrovascular disease	28 (5%)	8 (2%)	20 (8%)	**<0.01**	9 (3%)	19 (6%)	0.22
COPD	26 (4%)	4 (1%)	22 (9%)	**<0.01**	9 (3%)	17 (5%)	0.35
Chronic bronchitis	16 (3%)	7 (2%)	9 (4%)	0.18	4 (2%)	12 (4%)	0.13
Chronic liver disease	11 (2%)	4 (1%)	7 (3%)	0.11	4 (2%)	7 (2%)	0.62
Digestive disease	8 (1%)	3 (1%)	5 (2%)	0.19	4 (2%)	4 (1%)	0.71
Tuberculosis	6 (1%)	3 (1%)	3 (1%)	0.61	1 (0.4%)	5 (1%)	0.18

Supplementary Table 2. Mortality-related complications in non-survivors before their death.

	Total (N=102)	Female (N=37)	Male (N=65)	P-value
Any complication	102 (100%)	37 (100%)	65 (100%)	1.0
Respiratory failure	52 (51.0%)	18 (48.6%)	34 (52.3%)	0.72
Acute respiratory distress syndrome	29 (28.4%)	9 (24.3%)	20 (30.8%)	0.49
Multiple organ failure	28 (27.5%)	11 (29.7%)	17 (26.2%)	0.70
Shock	23 (22.5%)	9 (24.3%)	14 (21.5%)	0.75
Hypoxia	14 (13.7%)	6 (16.2%)	8 (12.3%)	0.58
Coma	9 (8.8%)	2 (5.4%)	7 (10.8%)	0.36
Hepatic insufficiency	9 (8.8%)	3 (8.1%)	6 (9.2%)	0.85
Fluid and electrolyte balance	9 (8.8%)	4 (10.8%)	5 (7.7%)	0.59
Renal insufficiency	8 (7.8%)	4 (10.8%)	4 (6.2%)	0.40
Heart failure	7 (6.9%)	4 (10.8%)	3 (4.6%)	0.23
Acute myocardial injury	5 (4.9%)	0 (0.0%)	5 (7.7%)	0.08
Acidosis	4 (3.9%)	2 (5.4%)	2 (3.1%)	0.56
Sepsis	3 (2.9%)	0 (0.0%)	3 (4.6%)	0.18
Disseminated intravascular coagulation	3 (2.9%)	0 (0.0%)	3 (4.6%)	0.18

Supplementary Table 3.1. Comparisons of baseline characteristics in the original sample and the propensity score-matched sample by gender.

	Original sample			Matched sample		
	**Female(n=184)**	**Male(n=215)**	**P-value**	**Female(n=157)**	**Male(n=157)**	**P-value**
Age, median (IQR)	66 (56.3 to 73)	67 (58 to 74)	0.37	65 (55.5 to 73)	67 (57 to 74)	0.29
**Region**						
China	170 (92.4%)	196 (91.2%)	0.66	149 (94.9%)	145 (92.4%)	0.36
Europe/North America	14 (7.6%)	19 (8.8%)		8 (5.1%)	12 (7.6%)	
**Signs or symptoms**						
Fever	152 (82.6%)	174 (80.9%)	0.67	130 (82.8%)	129 (82.2%)	0.88
Cough	117 (63.6%)	139 (64.7%)	0.83	97 (61.8%)	100 (63.7%)	0.73
Shortness of breath	57 (31.0%)	92 (42.8%)	0.02	54 (34.4%)	56 (35.7%)	0.81
Fatigue	46 (25.0%)	61 (28.4%)	0.45	44 (28.0%)	43 (27.4%)	0.90
Myalgia	29 (15.8%)	29 (13.5%)	0.52	21 (13.4%)	23 (14.6%)	0.75
Chills	24 (13.0%)	39 (18.1%)	0.16	23 (14.6%)	27 (17.2%)	0.54
Diarrhea	28 (15.2%)	31 (14.4%)	0.82	22 (14.0%)	23 (14.6%)	0.87
Headache	16 (8.7%)	15 (7.0%)	0.52	12 (7.6%)	13 (8.3%)	0.84
Nausea	11 (6.0%)	18 (8.4%)	0.36	10 (6.4%)	11 (7.0%)	0.82
Appetite loss	25 (13.6%)	32 (14.9%)	0.71	22 (14.0%)	24 (15.3%)	0.75
**Comorbidity**						
Any comorbidity	114 (62.0%)	141 (65.6%)	0.45	93 (59.2%)	91 (58.0%)	0.82
Hypertension	75 (40.8%)	88 (40.9%)	0.97	62 (39.5%)	62 (39.5%)	1.00
Diabetes mellitus	50 (27.2%)	53 (24.7%)	0.57	41 (26.1%)	35 (22.3%)	0.43
Cardiovascular disease	30 (16.3%)	38 (17.7%)	0.72	20 (12.7%)	25 (15.9%)	0.42
Cerebrovascular disease	9 (4.9%)	16 (7.4%)	0.30	7 (4.5%)	10 (6.4%)	0.45
Chronic liver disease	3 (1.6%)	6 (2.8%)	0.52	3 (1.9%)	1 (0.6%)	0.63
Digestive disease	4 (2.2%)	4 (1.9%)	1.00	3 (1.9%)	1 (0.6%)	0.63
Cancer	4 (2.2%)	16 (7.4%)	0.02	4 (2.5%)	2 (1.3%)	0.68
Chronic bronchitis	4 (2.2%)	12 (5.6%)	0.08	4 (2.5%)	4 (2.5%)	1.00
COPD	3 (1.6%)	8 (3.7%)	0.20	3 (1.9%)	2 (1.3%)	1.00
Tuberculosis	1 (0.5%)	5 (2.3%)	0.22	1 (0.6%)	1 (0.6%)	1.00

IQR: Interquartile range.

Supplementary Table 3.2. Comparisons of baseline characteristics in the original sample and the propensity score-matched sample by age.

	Original sample			Matched sample		
	<70 year (n=235)	≥70 year (n=164)	P-value	<70 year (n=129)	≥70 year (n=129)	P-value
Male	123 (52.3%)	92 (56.1%)	0.46	56 (43.4%)	69 (53.5%)	0.11
**Region**						
China	221 (94.0%)	145 (88.4%)		126 (97.6%)	119 (92.2%)	
European region	9 (3.9%)	11 (6.7%)	0.12	1 (0.8%)	8 (6.2%)	0.059
North America	5 (2.1%)	8 (4.9%)		2 (1.6%)	2 (1.6%)	
**Signs or symptoms**						
Fever	194 (82.6%)	132 (80.5%)	0.60	105 (81.4%)	109 (84.5%)	0.51
Cough	159 (67.7%)	97 (59.1%)	0.08	80 (62.0%)	80 (62.0%)	1.00
Shortness of breath	87 (37.0%)	62 (37.8%)	0.87	41 (31.8%)	45 (34.9%)	0.60
Fatigue	62 (26.4%)	45 (27.4%)	0.82	30 (23.3%)	38 (29.5%)	0.26
Myalgia	37 (15.7%)	21 (12.8%)	0.41	19 (14.7%)	20 (15.5%)	0.86
Chills	40 (17.0%)	23 (14.0%)	0.42	15 (11.6%)	19 (14.7%)	0.46
Diarrhea	36 (15.3%)	23 (14.0%)	0.72	24 (18.6%)	21 (16.3%)	0.62
Headache	20 (8.5%)	11 (6.7%)	0.51	11 (8.5%)	10 (7.8%)	0.82
Nausea	19 (8.1%)	10 (6.1%)	0.45	8 (6.2%)	8 (6.2%)	1.00
Appetite loss	28 (11.9%)	29 (17.7%)	0.11	14 (10.9%)	26 (20.2%)	0.06
**Comorbidity**						
Any comorbidity	127 (54.0%)	128 (78.0%)	9.0×10^{-7}	89 (69.0%)	93 (72.1%)	0.59
Hypertension	70 (29.8%)	93 (56.7%)	7.4×10^{-8}	60 (46.5%)	61 (47.3%)	0.90
Diabetes mellitus	49 (20.9%)	54 (32,9%)	0.007	42 (32.6%)	42 (32.6%)	1.00
Cardiovascular disease	23 (9.8%)	45 (27.4%)	4.0×10^{-6}	18 (14.0%)	18 (14.0%)	1.00
Cerebrovascular disease	7 (3.0%)	18 (11.0%)	0.001	5 (3.9%)	4 (3.1%)	1.00
Chronic liver disease	7 (3.0%)	2 (1.2%)	0.32	3 (2.3%)	1 (0.8%)	0.62
Digestive disease	1 (0.4%)	7 (4.3%)	0.01	1 (0.8%)	1 (0.8%)	1.00
Cancer	8 (3.4%)	12 (7.3%)	0.08	3 (2.3%)	9 (7.0%)	0.08
Chronic bronchitis	6 (2.6%)	10 (6.1%)	0.08	2 (1.6%)	8 (6.2%)	0.05
COPD	4 (1.7%)	7 (4.3%)	0.13	3 (2.3%)	3 (2.3%)	1.00
Tuberculosis	5 (2.1%)	1 (0.6%)	0.41	4 (3.1%)	0 (0.0%)	0.12

Supplementary Table 3.3. Comparisons of baseline characteristics in the original sample and the propensity score-matched sample by shortness of breath.

	Original sample			Matched sample		
	Shortness of breath (n=149)	Non-Shortness of breath (n=250)	P-value	Shortness of breath (n=133)	Non-Shortness of breath (n=133)	P-value
Male	92 (61.7%)	123 (49.2%)	0.02	81 (60.9%)	78 (58.6%)	0.71
Age, median (IQR)	67 (58 to 73.5)	66 (56 to 74)	0.29	66 (58 to 73)	64 (55 to 73)	0.12
**Region**						
China	129 (86.6%)	237 (94.8%)	0.004	123 (92.5%)	120 (90.2%)	0.51
Europe/North America	20 (13.4%)	13 (5.2%)		10 (7.5%)	13 (9.8%)	
**Signs or symptoms**						
Fever	120 (80.5%)	206 (82.4%)	0.64	110 (82.7%)	109 (82.0%)	0.87
Cough	100 (67.1%)	156(62.4%)	0.34	92 (69.2%)	81 (60.9%)	0.16
Fatigue	36 (24.2%)	71 (28.4%)	0.36	34 (25.6%)	37 (27.8%)	0.68
Myalgia	27 (18.1%)	31 (12.4%)	0.12	26 (19.5%)	24 (18.0%)	0.75
Chills	31 (20.8%)	32 (12.8%)	0.03	26 (19.5%)	25 (18.8%)	0.88
Diarrhea	24 (16.1%)	35 (14.0%)	0.57	21 (15.8%)	17 (12.8%)	0.48
Headache	12 (8.1%)	19 (7.6%)	0.87	10 (7.5%)	10 (7.5%)	1.00
Nausea	11 (7.4%)	18 (7.2%)	0.95	10 (7.5%)	8 (6.0%)	0.63
Appetite loss	19 (12.8%)	38 (15.2%)	0.50	18 (13.5%)	17 (12.8%)	0.86
**Comorbidity**						
Any comorbidity	100 (67.1%)	155 (62.0%)	0.30	84 (63.2%)	83 (62.4%)	0.90
Hypertension	59 (39.6%)	104 (41.6%)	0.69	50 (37.6%)	51 (38.3%)	0.90
Diabetes mellitus	42 (28.2%)	61 (24.4%)	0.40	37 (27.8%)	33 (24.8%)	0.58
Cardiovascular disease	35 (23.5%)	33 (13.2%)	0.008	23 (17.3%)	18 (13.5%)	0.40
Cerebrovascular disease	12 (8.1%)	13 (5.2%)	0.26	9 (6.8%)	3 (2.3%)	0.08
Chronic liver disease	2 (1.3%)	7 (2.8%)	0.49	2 (1.5%)	2 (1.5%)	1.00
Digestive disease	4 (2.7%)	4 (1.6%)	0.48	4 (3.0%)	0 (0.0%)	0.12
Cancer	6 (4.0%)	14 (5.6%)	0.49	6 (4.5%)	6 (4.5%)	1.00
Chronic bronchitis	6 (4.0%)	10 (4.0%)	0.99	6 (4.5%)	4 (3.0%)	0.52
COPD	9 (6.0%)	2 (0.8%)	0.003	1 (0.8%)	2 (1.5%)	1.00
Tuberculosis	1 (0.7%)	5 (2.0%)	0.42	1 (0.8%)	4 (3.0%)	0.37

IQR: interquartile range.

Supplementary Table 3.4. Comparisons of baseline characteristics in the original sample and the propensity score-matched sample by any comorbidity.

	Original sample			Matched sample		
	Comorbidity (n=255)	Non-comorbidity (n=144)	P-value	Comorbidity (n=123)	Non-comorbidity (n=123)	P-value
Male	141 (55.3%)	74 (51.4%)	0.45	64 (52.0%)	63 (51.2%)	0.90
Age, median (IQR)	70 (62 to 76)	62 (50 to 69.8)	2.5×10^{-10}	64 (56 to 70)	63 (57 to 70)	0.92
**Region**						
China	223 (87.5%)	143 (99.3%)	3.6×10^{-5}	121 (98.4%)	122 (99.2%)	1.00
Europe/North America	32 (12.5%)	1 (0.7%)		2 (1.6%)	1 (0.8%)	
**Signs or symptoms**						
Fever	204 (80.0%)	122 (84.7%)	0.24	104 (84.6%)	105 (85.4%)	0.86
Cough	159 (62.4%)	97 (67.4%)	0.32	79 (64.2%)	82 (66.7%)	0.69
Shortness of breath	100 (39.2%)	49 (34.0%)	0.30	44 (35.8%)	46 (37.4%)	0.79
Fatigue	67 (26.3%)	40 (27.8%)	0.75	35 (28.5%)	34 (27.6%)	0.89
Myalgia	34 (13.3%)	24 (16.7%)	0.36	19 (15.4%)	22 (17.9%)	0.61
Chills	35 (13.7%)	28 (19.4%)	0.13	18 (14.6%)	23 (18.7%)	0.39
Diarrhea	38 (14.9%)	21 (14.6%)	0.93	20 (16.3%)	19 (15.4%)	0.86
Headache	18 (7.1%)	13 (9.0%)	0.48	11 (8.9%)	9 (7.3%)	0.64
Nausea	18 (7.1%)	11 (7.6%)	0.83	12 (9.8%)	8 (6.5%)	0.35
Appetite loss	35 (13.7%)	22 (15.3%)	0.67	19 (15.4%)	18 (14.6%)	0.86

IQR: interquartile range.

Supplementary Table 3.5. Comparisons of baseline characteristics in the original sample and the propensity score-matched sample by cardiovascular disease.

	Original sample			Matched sample		
	Cardiovascular disease (n=68)	Non-cardiovascular disease (n=331)	P-value	Cardiovascular disease (n=58)	Non-cardiovascular disease (n=58)	P-value
Male	38 (55.9%)	177 (53.5%)	0.72	31 (53.4%)	34 (58.6%)	0.58
Age, median (IQR)	73.5 (67 to 82)	65 (56 to 72)	2.1×10^{-10}	73 (66 to 80)	73 (68 to 81)	0.66
**Region**						
China	55 (80.9%)	311 (94.0%)	3.6×10^{-4}	51 (87.9%)	55 (94.8%)	0.19
Europe/North America	13 (19.1%)	20 (6.0%)		7 (12.1%)	3 (5.2%)	
**Signs or symptoms**						
Fever	50 (73.5%)	276 (83.4%)	0.06	45 (77.6%)	45 (77.6%)	1.00
Cough	41 (60.3%)	215 (65.0%)	0.47	36 (62.1%)	28 (48.3%)	0.14
Shortness of breath	35 (51.5%)	114 (34.4%)	0.008	25 (43.1%)	26 (44.8%)	0.85
Fatigue	14 (20.6%)	93 (28.1%)	0.20	13 (22.4%)	15 (25.9%)	0.66
Myalgia	6 (8.8%)	52 (15.7%)	0.14	6 (10.3%)	7 (12.1%)	0.77
Chills	11 (16.2%)	52 (15.7%)	0.92	10 (17.2%)	8 (13.8%)	0.61
Diarrhea	9 (13.2%)	50 (15.1%)	0.69	8 (13.8%)	8 (13.8%)	1.00
Headache	5 (7.4%)	26 (7.9%)	0.89	5 (8.6%)	4 (6.9%)	1.00
Nausea	3 (4.4%)	26 (7.9%)	0.44	3 (5.2%)	3 (5.2%)	1.00
Appetite loss	5 (7.4%)	52 (15.7%)	0.07	5 (8.6%)	6 (10.3%)	0.75
**Comorbidity**						
Any comorbidity	68 (100.0%)	187 (56.5%)	1.0×10^{-11}	58 (100.0%)	58 (100.0%)	–
Hypertension	43 (63.2%)	120 (36.3%)	3.7×10^{-5}	36 (62.1%)	34 (58.6%)	0.70
Diabetes mellitus	24 (35.3%)	79 (23.9%)	0.05	20 (34.5%)	27 (46.6%)	0.19
Cerebrovascular disease	10 (14.7%)	15 (4.5%)	0.004	6 (10.3%)	8 (13.8%)	0.57
Chronic liver disease	2 (2.9%)	7 (2.1%)	0.65	2 (3.4%)	2 (3.4%)	1.00
Digestive disease	3 (4.4%)	5 (1.5%)	0.14	3 (5.2%)	3 (5.2%)	1.00
Cancer	5 (7.4%)	15 (4.5%)	0.36	5 (8.6%)	4 (6.9%)	1.00
Chronic bronchitis	5 (7.4%)	11 (3.3%)	0.17	4 (6.9%)	4 (6.9%)	1.00
COPD	8 (11.8%)	3 (0.9%)	5.3×10^{-5}	3 (5.2%)	2 (3.4%)	1.00
Tuberculosis	2 (2.9%)	4 (1.2%)	0.27	2 (3.4%)	0 (0.0%)	0.50

IQR: interquartile range.

Supplementary Table 3.6. Comparisons of baseline characteristics in the original sample and the propensity score matched sample by cerebrovascular disease.

	Original sample			Matched sample		
	Cerebrovascular disease (n=25)	Non-cerebrovascular disease (n=374)	P-value	Cerebrovascular disease (n=25)	Non-cerebrovascular disease (n=25)	P-value
Male	16 (64.0%)	199 (53.2%)	0.30	16 (64.0%)	9 (36.0%)	0.05
Age, median (IQR)	78 (65.5 to 84)	66 (57 to 73)	2.0×10^{-4}	78 (65.5 to 84)	74 (65.5 to 83)	0.83
**Region**						
China	21 (84.0%)	345 (92.2%)	0.14	21 (84.0%)	25 (100%)	0.11
Europe/North America	4 (16.0%)	29 (7.8%)		4 (16.0%)	0 (0.0%)	
**Signs or symptoms**						
Fever	19 (76.0%)	307 (82.1%)	0.43	19 (76.0%)	18 (72.0%)	0.75
Cough	13 (52.0%)	243 (65.0%)	0.19	13 (52.0%)	13 (52.0%)	1.00
Shortness of breath	12 (48.0%)	137 (36.6%)	0.26	12 (48.0%)	8 (32.0%)	0.25
Fatigue	6 (24.0%)	101 (27.0%)	0.74	6 (24.0%)	6 (24.0%)	1.00
Myalgia	4 (16.0%)	54 (14.4%)	0.77	4 (16.0%)	2 (8.0%)	0.67
Chills	4 (16.0%)	59 (15.8%)	1.00	4 (16.0%)	0 (0.0%)	0.11
Diarrhea	2 (8.0%)	57 (15.2%)	0.56	2 (8.0%)	4 (16.0%)	0.67
Headache	3 (12.0%)	28 (7.5%)	0.43	3 (12.0%)	1 (4.0%)	0.61
Nausea	4 (16.0%)	25 (6.7%)	0.10	4 (16.0%)	1 (4.0%)	0.35
Appetite loss	3 (12.0%)	54 (14.4%)	1.00	3 (12.0%)	2 (8.0%)	1.00
**Comorbidity**						
Any comorbidity	25 (100.0%)	230 (61.5%)	1.0×10^{-4}	25 (100.0%)	25 (100.0%)	–
Hypertension	18 (72.0%)	145 (38.8%)	0.001	18 (72.0%)	20 (80.0%)	0.51
Diabetes mellitus	10 (40.0%)	93 (24.9%)	0.09	10 (40.0%)	14 (56.0%)	0.26
Cardiovascular disease	10 (40.0%)	58 (15.5%)	0.004	10 (40.0%)	11 (44.0%)	0.77
Chronic liver disease	0 (0.0%)	9 (2.4%)	1.00	0 (0.0%)	0 (0.0%)	–
Digestive disease	2 (8.0%)	6 (1.6%)	0.08	2 (8.0%)	0 (0.0%)	0.49
Cancer	2 (8.0%)	18 (4.8%)	0.36	2 (8.0%)	1 (4.0%)	1.00
Chronic bronchitis	2 (8.0%)	14 (3.7%)	0.27	2 (8.0%)	1 (4.0%)	1.00
COPD	1 (4.0%)	10 (2.7%)	0.51	1 (4.0%)	0 (0.0%)	1.00
Tuberculosis	0 (0.0%)	6 (1.6%)	1.00	0 (0.0%)	1 (4.0%)	1.00

IQR: interquartile range.

Supplementary Table 3.7. Comparisons of baseline characteristics in the original sample and the propensity score matched sample by COPD.

	Original sample			Matched sample		
	COPD (n=11)	Non-COPD (n=388)	P-value	COPD (n=9)	Non-COPD (n=9)	P-value
Male	8 (72.7%)	207 (53.4%)	0.20	8 (88.9%)	5 (55.6%)	0.29
Age, median (IQR)	78 (66 to 84)	66 (57 to 73)	0.01	75 (65 to 81)	72 (67 to 82)	0.93
**Region**						
China	7 (63.6%)	359 (92.5%)	0.009	7 (77.8%)	7 (77.8%)	1.00
Europe/North America	4 (36.4%)	29 (7.5%)		2 (22.2%)	2 (22.2%)	
**Signs or symptoms**						
Fever	6 (54.5%)	320 (82.5%)	0.03	6 (66.7%)	6 (66.7%)	1.00
Cough	7 (63.6%)	249 (64.2%)	1.00	6 (66.7%)	6 (66.7%)	1.00
Shortness of breath	9 (81.8%)	140 (36.1%)	0.003	7 (77.8%)	6 (66.7%)	1.00
Fatigue	2 (18.2%)	105 (27.1%)	0.73	2 (22.2%)	3 (33.3%)	1.00
Myalgia	1 (9.1%)	57 (14.7%)	1.00	1 (11.1%)	1 (11.1%)	1.00
Chills	2 (18.2%)	61 (15.7%)	0.69	2 (22.2%)	0 (0.0%)	0.47
Diarrhea	3 (27.3%)	56 (14.4%)	0.21	3 (33.3%)	1 (11.1%)	0.58
Headache	2 (18.2%)	29 (7.5%)	0.21	2 (22.2%)	1 (11.1%)	1.00
Nausea	1 (9.1%)	28 (7.2%)	0.57	1 (11.1%)	0 (0.0%)	1.00
Appetite loss	1 (9.1%)	56 (14.4%)	1.00	1(11.1%)	1 (11.1%)	1.00
**Comorbidity**						
Any comorbidity	11 (100%)	244 (62.9%)	0.009	9 (100%)	8 (88.9%)	1.00
Hypertension	6 (54.5%)	157 (40.5%)	0.37	4 (44.4%)	6 (66.7%)	0.64
Diabetes mellitus	5 (45.5%)	98 (25.3%)	0.16	4 (44.4%)	4 (44.4%)	1.00
Cardiovascular disease	8 (72.7%)	60 (15.5%)	5.3×10^{-5}	6 (66.7%)	7 (77.8%)	1.00
Cerebrovascular disease	1 (9.1%)	24 (6.2%)	0.51	1 (11.1%)	2 (22.2%)	1.00
Chronic liver disease	0 (0.0%)	9 (2.3%)	1.00	0 (0.0%)	0 (0.0%)	–
Digestive disease	0 (0.0%)	8 (2.1%)	1.00	0 (0.0%)	0 (0.0%)	–
Cancer	1 (9.1%)	19 (4.9%)	0.44	1 (11.1%)	0 (0.0%)	1.00
Chronic bronchitis	1 (9.1%)	15 (3.9%)	0.37	1 (11.1%)	0 (0.0%)	1.00
Tuberculosis	0 (0.0%)	6 (1.5%)	1.00	0 (0.0%)	0 (0.0%)	–

IQR: interquartile range.

Supplementary Table 4. Retrieved online records of COVID-19 patients in China, European regions, and North America.

Country	>5 dead cases by 2020/04/30 #	Retrieved records in total *	Excluded records &	Records in our analysis
China	Yes	416	1	415
**European regions**				
Albania	Yes	5	0	5
Andorra	Yes	1	1	0
Austria	Yes	20	0	20
Belarus	Yes	1	1	0
Belgium	Yes	15	4	11
Bosnia and Herzegovina	Yes	2	2	0
Bulgaria	Yes	6	0	6
Croatia	Yes	7	1	6
Cyprus	Yes	3	3	0
Czechia	Yes	4	4	0
Denmark	Yes	3	3	0
Estonia	Yes	3	3	0
Finland	Yes	0	0	0
France	Yes	62	4	58
Georgia	Yes	6	4	2
Germany	Yes	49	0	49
Greece	Yes	15	0	15
Hungary	Yes	15	0	15
Iceland	Yes	3	3	0
Ireland	Yes	8	0	8
Italy	Yes	56	3	53
Latvia	Yes	0	0	0
Liechtenstein	No	-	-	-
Lithuania	Yes	6	0	6
Luxembourg	Yes	1	1	0
Malta	No	-	-	-
Monaco	No	-	-	-
Montenegro	Yes	1	1	0
Netherlands	Yes	9	0	9
North Macedonia	Yes	4	4	0
Norway	Yes	1	1	0
Poland	Yes	24	0	24
Portugal	Yes	4	4	0
Republic of Moldova	Yes	2	2	0
Romania	Yes	7	0	7
Russian Federation	Yes	6	1	5
San Marino	Yes	1	1	0
Serbia	Yes	7	0	7
Slovakia	Yes	1	1	0

Slovenia	Yes	3	3	0
Spain	Yes	201	76	125
Sweden	Yes	1	1	0
Switzerland	Yes	4	4	0
The United Kingdom	Yes	35	0	35
Ukraine	Yes	2	2	0
**North America**				
United States of America	Yes	191	24	167
Antigua and Barbuda	No	-	-	0
Bahamas	Yes	1	1	0
Barbados	Yes	1	1	0
Belize	No	-	-	0
Canada	Yes	5	2	3
Costa Rica	Yes	2	2	0
Cuba	Yes	6	0	6
Dominica	No	-	-	0
Dominican Republic	Yes	4	4	0
El Salvador	Yes	0	0	0
Grenada	No	-	-	0
Guatemala	Yes	1	1	0
Haiti	Yes	2	2	0
Honduras	Yes	4	4	0
Jamaica	Yes	2	2	0
Mexico	Yes	18	0	18
Nicaragua	Yes	1	1	0
Panama	Yes	3	3	0
Saint Kitts and Nevis	No	-	-	0
Saint Lucia	No	-	-	0
Saint Vincent and the Grenadines	No	-	-	0
Trinidad and Tobago	Yes	2	2	0
**Total**		1267	192	1075

#: "Yes" indicates a country with ≥5 dead cases by 2020/04/30. The number of dead cases was retrieved based on the situation report of COVID-19 from the WHO (date: April 30, 2020).
*: Total number of retrieved online records from each country.
&: Records were excluded if they failed to fulfill four selection criteria:
(i) Patients did not receive remdesivir;
(ii) Patients did not receive dexamethasone;
(iii) Patients cases were hospitalized after May 1 and had missing data of therapy;
(iv) Countries with limited number of COVID-19 cases in the dataset (<5).
