# Supplementary material for: Mortality risk of COVID-19 in elderly males with comorbidities: a multi-country study
Source: Aging (Albany NY). 2020 Dec 31;13(1):27–60. doi: 10.18632/aging.202456 (PMC7835001; doi:10.18632/aging.202456)
Supplement: Supplementary Method 1 [file aging-13-202456-s003.pdf]

Supplementary Method

Supplementary Method 1: Discharge criteria of COVID-19 patients

Based on the New Coronavirus Diagnosis and Treatment Guidelines in China, patients were discharged if they fulfilled all three conditions:

(i) At least two consecutive results of undetectable SARS-CoV-2 in throat swab samples which were collected at least 24 hours apart;
(ii) Clinical remission of respiratory symptoms and fever for at least three consecutive days;

(iii) Substantial improvement of both lungs based on computed tomography.

All discharged patients remained on home isolation for another 14 days.
